# The Relationship Between Child Maltreatment and Dispositional Envy and the Mediating Effect of Self-Esteem and Social Support in Young Adults

**DOI:** 10.3389/fpsyg.2018.01054

**Published:** 2018-07-20

**Authors:** Yanhui Xiang, Weixin Wang, Fang Guan

**Affiliations:** ^1^Department of Psychology, Hunan Normal University, Changsha, China; ^2^Cognition and Human Behavior Key Laboratory of Hunan Province, Hunan Normal University, Changsha, China; ^3^School of Psychology, South China Normal University, Guangzhou, China; ^4^Center for Studies of Psychological Application, South China Normal University, Guangzhou, China

**Keywords:** child maltreatment, dispositional envy, self-esteem, perceived social support, structural equation model

## Abstract

As child maltreatment is a common social problem worldwide, the present study explores the relationship between child maltreatment and dispositional envy and the mediating effects of self-esteem and social support in this relationship. Data were collected from 426 Chinese college students (*M* age = 20.63, *SD* = 1.85), using the Child Abuse Scale, Dispositional Envy Scale, Rosenberg Self-Esteem Scale, and Multi-Dimensional Scale of Perceived Social Support. The results show that self-esteem plays a partially mediating role in the association between child maltreatment and envy. In addition, sequential mediation analyses have further revealed the maltreatment effect of envy, through social support and self-esteem. Further, a multiple-group analysis has shown that men with high child maltreatment scores tend to have lower levels of social support than women. These results provide an important reference for revealing how maltreatment early in life effects social emotion in adulthood, particularly dispositional envy. They may provide a valuable resource for psychological interventions targeting people of both genders who are victims of child maltreatment.

## Introduction

Child maltreatment is a common problem worldwide (Butchart et al., 2006), with devastating short- and long-term consequences for children (Burgess et al., 2012). Accordingly, it has drawn considerable multidisciplinary attention. Child maltreatment includes emotional abuse, physical abuse, sexual abuse, emotional neglect, and physical neglect. Numerous studies have found that child maltreatment has far-reaching impacts on mental health. Previous studies have found that adolescents who have experienced maltreatment in childhood are more likely to develop psychiatric disorders, such as depression, anxiety, and social withdrawal (Dehon and Weems, 2010; Leslie et al., 2010). This may be because, as previous studies have confirmed (Brassard et al., 1991; Al Odhayani et al., 2013), childhood abuse affects the normal development of physical and mental health. Social emotion is extremely important for psychological development. Among all types of social emotion, envy is both typical and common, with widespread effects, especially for individual mental health (Smith and Combs, 2008). Envy is defined as a painful emotion characterized by feelings of inferiority, hostility, and resentment, usually caused by lacking the good qualities, achievements, or possessions of others in social comparisons. People who experience envy long to either obtain these advantages or see others lose them (Parrott and Smith, 1993). However, at present, few studies have explored whether and in what way child maltreatment affects individual envy.

### The Possible Relationship Between Child Maltreatment and Envy

Although the relationship between child maltreatment and envy has not been explicitly studied, there is considerable evidence to guide reasonable hypotheses. Individuals who experience childhood maltreatment may develop strong social pain (the painful feelings that follow from social rejection, exclusion, or being devalued by desired partners or groups, Eisenberger, 2015). Such individuals may feel that their basic emotional needs have been ignored, that they are isolated and cut off from caregivers, or that their siblings are favored. Social pain is the core characteristic of both dispositional and episodic envy. Previous studies using brain imaging have found that any kind of envy will elicit activity in the anterior cingulate cortex (Takahashi et al., 2009; Xiang et al., 2016), which is closely related to social pain (Eisenberger et al., 2003). Takahashi et al. (2009) have suggested that envy may be a form of social pain in the self, accompanied by feelings of being excluded from self-related fields. It has also been found that children who experience maltreatment during childhood tend to experience higher levels of shame than control participants in studies (Fossum and Mason, 1986; Steele, 1987; Feiring et al., 1996; Claesson and Sohlberg, 2002). Shame is a factor frequently cited as a key component of envy because shame is a typical response to having a devalued self; it always follows the envy-causing inferiority that results from an upward social comparison (Smith and Kim, 2007). Furthermore, the experience of unmet physical needs (physical neglect) or caretakers’ failure to provide adequate affection or emotional support (emotional neglect) may lead to material and emotional deprivation. Subjects may desperately want to be loved or overly concerned with what others have that they themselves do not have, such as food, clothes, toys, substance, or money. They may generalize this feeling of deprivation to other aspects of life, making themselves prone to feelings of envy. Based on the evidence above, the present study hypothesizes that child maltreatment may be closely associated with envy. Exploring this connection could enhance our understanding of the relationship between child maltreatment, envy, and social emotion in general. This study therefore begins by investigating whether and how child maltreatment affects envy.

### Child Maltreatment, Self-Esteem, Perceived Social Support, and Envy

If the hypothesis about the relationship between child maltreatment and envy is correct, a further discussion of the mechanisms or processes underlying this relationship will be necessary.

A likely candidate to mediate the association between child maltreatment and envy is self-esteem. Self-esteem refers to one’s general sense of one’s own value or worth (Rosenberg, 1979). Some scholars have suggested that parents play a critical role in the development of children’s self-esteem (Harter, 2008; Shaffer and Kipp, 2010). Parents whose responses are warm and supportive, providing a positive family atmosphere, are valuable in shaping self-esteem in children and adolescents (Shaffer and Kipp, 2010). Conversely, negative parental feedback and the lack of emotional interaction (e.g., psychological maltreatment subtypes) can be detrimental to the development of self-esteem. Some evidence supports this hypothesis. For instance, numerous studies have revealed a strong connection between psychological maltreatment and low self-esteem (Gross and Keller, 1992; Stein et al., 2002; Karakuş, 2012). In other words, individuals who have been maltreated may develop low self-esteem in childhood and adulthood (Miller-Perrin and Perrin, 2012). Some studies have further indicated that envy is associated with low self-esteem (Salovey and Rodin, 1991; Rentzsch et al., 2015). Thus, child maltreatment may be associated with higher levels of envy, due to reduced self-esteem.

Perceived social support may be another promising mediator of this relationship. Crouch et al. (2001) have found that childhood physical abuse and perceived social support tend to covary. Specifically, the more physical abuse children have suffered, the lower the levels of early support they tend to receive. Lower levels of early childhood support have a negative influence on current perceived levels of support, which, in turn, further expose children to a higher risk of physical abuse (Crouch et al., 2001). In line with this, several studies have found that children who suffer from neglect and/or emotional abuse have peer difficulties and poor social skills (Kaufman and Cicchetti, 1989; Manly et al., 1994, 2001; De Paúl and Arruabarrena, 1995; Bolger et al., 1998; Kinard, 1999; Finzi et al., 2002, 2003; Kim and Cicchetti, 2009). This may be due to the disorganized attachment patterns acquired through caregiver relationships (Rutter, 1995; Bretherton, 1997; McCain et al., 2007), where the attachment patterns continue to influence ongoing and future relationships with significant others (Cicchetti et al., 2006). Childhood physical abuse, for example, has been found to be negatively associated with perceptions of support from family and friends until adulthood (Runtz and Schallow, 1997; Crouch et al., 2001). Recent evidence has also revealed that abuse and neglect during childhood are generally related to lower levels of perceived social support in adulthood (Vranceanu et al., 2007; Sperry and Widom, 2013). Moreover, social support has a significant negative correlation with envy (Smith et al., 1988). This may be because individuals with lower levels of social support are more hostile or sensitive in comparing themselves socially, facilitating the experience of malicious envy. Similarly, higher levels of social support may be conducive to less hostile and more positive and optimistic social comparisons, which make it easier for people to experience benign envy. Based on this evidence, child maltreatment may be associated with higher levels of envy, due to impaired social support.

The relationship between self-esteem and perceived social support has been studied extensively. Perceived social support is a decisive factor in building high self-esteem (Rosenberg, 1979; Mruk, 2006). Numerous empirical studies have shown that perceived social support is significantly positively correlated with self-esteem (Hoffman et al., 1988; Tam et al., 2011; Kong et al., 2012b; Kong and You, 2013). This means that adequate social support, received from family, friends, or significant others, is conducive to high self-esteem. Perceived social support may therefore influence envy through the mediation of self-esteem.

### The Role of Gender

There is some evidence that gender plays an important role at different points along these hypothesized pathways. In one example of gender differences in perceived social support, girls benefit more than boys from the buffering effect of perceived social support on emotional and health outcomes (Windle, 1992). In single-parent families, boys may benefit more than girls from male involvement (Hetherington et al., 1998). When it comes to self-esteem, boys with low self-esteem develop emotional problems, such as depression, more easily than girls with low self-esteem (Abela and Payne, 2003). These findings may differ as result of methodological differences. They may also suggest that the role of gender is complex and variable, depending on the particular predictors and outcomes being evaluated. Given the mixed nature of the existing literature on gender differences within these pathways, we will use an exploratory approach to examine gender differences, proposing no specific hypotheses.

### The Current Study

Although no previous study has investigated the relationship between child maltreatment and dispositional envy, there is sufficient evidence, outlined above, to guide the generation of reasonable hypotheses. The goal of this study has been to test the concurrent mediation effects of perceived social support and self-esteem on the relationship between early child maltreatment and dispositional envy within personal and interpersonal frameworks. In view of the association between child maltreatment and perceived social support (Vranceanu et al., 2007; Sperry and Widom, 2013) and self-esteem (Gross and Keller, 1992; Stein et al., 2002; Karakuş, 2012) and the important role of self-esteem (Salovey and Rodin, 1991) and perceived social support in dispositional envy (Smith et al., 1988), we have predicted that perceived social support and self-esteem may act as mediators in the relationship. In addition, to further demonstrate the reliability of the final model, we have tested for differences between men and women through a supplementary analysis, based on previous studies (Sun and Kong, 2013; Kong et al., 2015).

In summary, the present study explores the mediating effects of self-esteem and perceived social support on the child maltreatment–envy relationship in a sample of Chinese college students. Two possible hypotheses were proposed: (1) Child maltreatment significantly predicts dispositional envy; (2) Self-esteem and perceived social support mediate the association between child maltreatment and dispositional envy.

## Materials and Methods

### Participants and Procedure

Through campus advertising, we randomly recruited 426 Chinese undergraduates from South China Normal University and Jinan University to participate in the current study; no data were excluded. Both universities are national key construction universities offering the similar educational resources. The basic demographic characteristics of the participants were as follows: 142 males and 284 females; mean age = 20.63 ± 1.85; age range = 18–26 years. Participants voluntarily completed a series of questionnaires in a classroom during a 40-min period; they received 40 RMB as compensation. The present study was approved by the Academic Committee of the School of Psychology at South China Normal University. All participants provided written, informed consent.

### Measures

#### Childhood Abuse Scale (CAS)

The CAS was developed by Bernstein et al. (1997). The original scale consists of 28 items, such as “I thought that my parents wished I had never been born” and “I had to wear dirty clothes.” The Chinese version of the CAS was revised by Zhao et al. (2005) and has good reliability and validity in Chinese samples. This scale has five subscales, including emotional abuse, physical abuse, sexual abuse, emotional neglect, and physical neglect. However, because sexual abuse is a sensitive subject in Chinese culture, we focused only on the other four subscales. Each item was scored on a 5-point Likert-type scale. The four subscales’ Cronbach’s alpha coefficients were as follows: 0.69 for emotional abuse, 0.77 for physical abuse, 0.79 for emotional neglect, and 0.61 for physical neglect in the present study.

#### Dispositional Envy Scale (DES)

The DES (Smith et al., 1999) consists of 8 items, such as “No matter what I do, envy always plagues me” and “I feel envy every day.” Each item was scored on a 5-point Likert-type scale. This scale has been used to investigate Chinese samples, and is reliable and valid (Xiang et al., 2016, 2017). In this study, the scale showed adequate internal reliability (α = 0.85).

#### Multi-Dimensional Scale of Perceived Social Support (MSPSS)

This scale was developed by Zimet et al. (1988), which has 12 items scored on a 7-point Likert-type scale, including items such as “My family really tries to help me.” This scale consists of three subscales: family support, friend support, and others’ support. The Chinese version of the MSPSS has been found to be a reliable and valid measurement in assessing social support in Chinese populations (Kong et al., 2012a,b; Zhao et al., 2013). In the present study, the Cronbach’s alpha coefficient for the MSPSS was 0.90 for the entire scale, 0.86 for family, 0.90 for friends, and 0.83 for others.

#### Rosenberg Self-Esteem Scale (RSES)

This scale was developed by Rosenberg (1965). It consists of 10 items scored on a 4-point Likert-type scale. An example of an item is “I take a positive attitude toward myself.” The Chinese version of the RSES has been demonstrated to be a reliable and valid measurement in assessing self-esteem in Chinese populations (Kong et al., 2012a,b; Zhao et al., 2013). In the present study, Cronbach’s alpha coefficient for the RSES was 0.90.

### Data Analysis

According to Anderson and Gerbing (1988), it is necessary to carry out a two-step procedure to analyze mediation effects. In order to test whether latent variables were represented by their indicators, the first step was to test the measurement model. In the second step, we further tested the structural model in AMOS 17.0. We then divided the MSPSS and the DES into two or three parcels to serve as indicators of the factors, using an item-to-construct balance approach (Little et al., 2002). To judge the model’s goodness-of-fit, we used chi-square statistics, a standardized root-mean-square residual (SRMR), a root-mean-square error of approximation (RMSEA), and a comparative fit index (CFI) as the indicators (Hu and Bentler, 1999). In addition, we used the Akaike information criterion (AIC) to judge the best-fit model (Akaike, 1987). The expected cross-validation index (ECVI) was used to evaluate potential for replication (Browne and Cudeck, 1992).

## Results

### Measurement Model

There are 4 latent factors (maltreatment, perceived social support, self-esteem, and envy) and 11 observed variables in this measurement model. The main measurement indexes were as follows: χ^2^ (36, 426) = 115.92, *p* < 0.001; SRMR = 0.0538; RMSEA = 0.060; CFI = 0.96. Furthermore, all of the factor loadings on the latent variables were significant (*p* < 0.05). The correlation analyses among maltreatment, envy, perceived social support, and self-esteem showed a significant correlation for each variable (see **Table 1**).

**Table 1:** Descriptive statistics and zero-order correlations for all variables.

Measure	*M*	*SD*	1	2	3	4
(1) Childhood maltreatment	31.40	7.99	1			
(2) MSPSS	62.49	11.17	−0.36^∗∗∗^	1		
(3) RSES	30.22	4.91	−0.39^∗∗∗^	0.43^∗∗∗^	1	
(4) Envy	17.49	6.07	0.38^∗∗∗^	−0.34^∗∗∗^	−0.67^∗∗∗^	1

*MSPSS, Multi-Dimensional Scale of Perceived Social Support; RSES, Rosenberg self-esteem scale. ^∗∗∗^p < 0.001.*

### Structural Model

**FIGURE 1:**
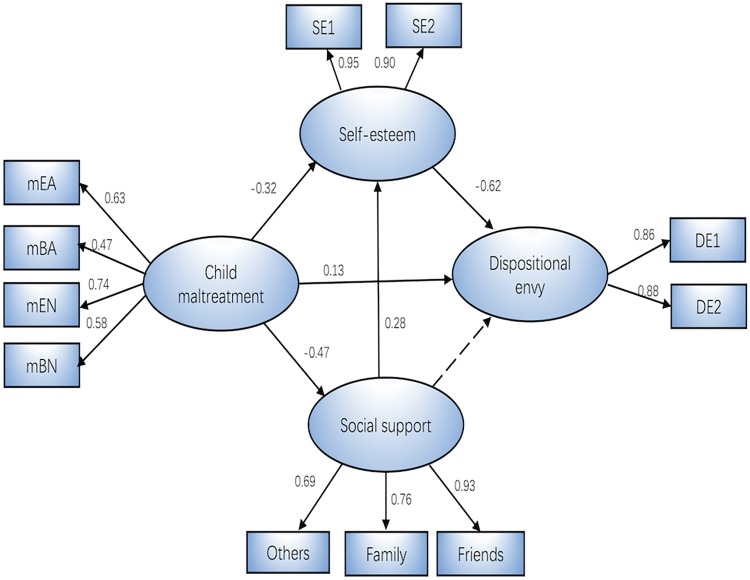
The mediation model (*N* = 426). Factor loadings are standardized. *SE1* and *SE2* are two parcels of the Rosenberg Self-Esteem Scale; *DE1* and *DE2* are two parcels of the Dispositional Envy Scale; *Others, Family,* and *Friends* are the subscales of the Multi-Dimensional Scale of Perceived Social Support, which are others’ support, family support, and friend support; *mEA, mBA, mEN,* and *mBN* are the subscales of the Childhood Abuse Scale, which are emotional abuse, body abuse, emotional neglect, and body neglect. All the path coefficients are significant at the 0.05 level.

In order to find the best model, we established three alternative models. First, we assessed a partially mediated model (Model 1) with two mediators and two direct paths from RSES and perceived social support to envy (**Table 2**). As shown in **Table 2**, the results revealed a good fit to the data, except that RMSEA was greater than 0.06. Maltreatment was found to have a significant effect on envy at the 0.10 level (*b* = 0.115, *p* = 0.083), while perceived social support did not have a significant effect on envy (*b* = −0.055, *p* = 0.236). Based on this analysis, we set the path from perceived social support to envy at zero to establish Model 2, where the indicators remained relatively constant. When we compared Models 1 and 2, the AIC value was smaller for Model 2. To determine the best model, according to previous research, we added a path from perceived social support to RSES (Model 3). The indicators from Model 3 were better than were those of Models 1 and 2, with a smaller χ^2^, AIC, and ECVI. Taken together, Model 3 was considered the best among those explored (**Figure 1**).

**Table 2:** Fit indices among competing models.

	*χ^2^*	*df*	RMSEA	SRMR	CFI	AIC	ECVI
Model 1	96.703	36	0.063	0.0557	0.971	156.708	0.369
Model 2	98.089	37	0.062	0.0579	0.971	156.089	0.367
Model 3	68.678	36	0.046	0.033	0.985	128.678	0.303

*N = 426. RMSEA, root-mean-square error of approximation; SRMR, standardized root-mean-square residual; CFI, comparative fit index; PGFI, parsimony goodness of fit index; PNFI, parsimony normed fit index; AIC, Akaike information criterion; ECVI, expected cross-validation index.*

During the preliminary process of assessing the structural model, we illustrated the important mediating role of RSES and perceived social support between maltreatment and envy. However, standard error estimates and confidence intervals require normally distributed data, and this condition can be difficult to satisfy. To further test the stability of the result, we therefore used a bootstrap estimate procedure to test the stability of such mediating mechanisms. The bootstrap procedure did not require data to be normally distributed (MacKinnon et al., 2004). Based on this procedure, if the confidence interval of the estimated path coefficient did not include zero, we could conclude that the mediating effect was significant. As shown in **Table 3**, the mediating effects were significant for all three paths. Maltreatment exerted a significant indirect effect on envy via perceived social support and RSES, while perceived social support influenced envy indirectly via RSES.

**Table 3:** Unstandardized indirect effects and 95% confidence intervals.

	Estimate	Lower	Upper
Maltreat-RSES-Envy	0.32^a^	0.17	0.50
Maltreat-Perceived social support-RSES	−0.24^a^	−0.42	−0.10
Perceived social support-RSES-Envy	−0.17^a^	−0.29	−0.08
Maltreat-perceived social support-RSES-Envy	0.16	0.06	0.29

*RSES, Rosenberg Self-Esteem Scale. ^a^Empirical 95% confidence interval does not overlap with zero.*

### Gender Differences

We found no statistically significant gender differences associated with maltreatment [*t*_(424)_ = 0.437, *p* = 0.662], RSES [*t*_(424)_ = 0.824, *p* = 0.411], or envy [*t*_(424)_ = −0.931, *p* = 0.353], but women scored significantly higher than men on perceived social support [*t*_(424)_ = −2.437, *p* = 0.015]. We therefore used a multi-group analysis to investigate gender differences in the path coefficients. Controlling for variables such as factor loadings, error variances, and structure covariances, we established two models. The first model allowed the structure coefficient of the two models to be estimated freely according to gender. The second model was controlled for the structure path coefficient to be equal. Results showed that this model was not significant, Δχ*^2^*(5, *N* = 426) = 3.980, *p* = 0.552, indicating that it was consistent across gender. In addition, we used critical ratios of differences (CRDs) as an indicator to investigate the difference in standard error across gender. If the CRD was greater than 1.96, that might suggest that the two variables would show a significant difference, at *p* < 0.05. We found that, for the path from maltreatment to perceived social support, the structural path showed a significant difference (CRD = 2.808, *p* < 0.05). Specifically, the path coefficient for men was β = −0.56, *p* < 0.001, while the path coefficient for women was β = −0.44, *p* < 0.001. In other words, child maltreatment had a far greater negative effect on perceived social support among men than among women.

## Discussion

This study explored the relationship between child maltreatment and envy, as well as the mediating role of self-esteem and perceived social support, among Chinese college students. As expected, child maltreatment was positively related to dispositional envy. In other words, people who have been maltreated in childhood are more likely to experience envy. Moreover, maltreatment can affect envy indirectly, via self-esteem or perceived social support and self-esteem. To our knowledge, this is the first study to explore the relationship between maltreatment and envy and the way in which this relationship is mediated by perceived social support and self-esteem.

As hypothesized, the direct effect of maltreatment on envy may be attributed to social pain. Previous studies have indicated that envy itself is a painful social emotion involving inferiority and resentment (Aristotle, 1991; Parrott and Smith, 1993); this finding has been demonstrated in brain imaging studies (Xiang et al., 2016, 2017). Social pain is the painful feeling that follows social exclusion. Undoubtedly, maltreatment by caregivers (including physical abuse, physical neglect, emotional abuse, and emotional neglect) is the most typical type of social rejection and a source of social pain in children. It may lead to feelings of envy in adulthood. In addition, child maltreatment can indirectly affect envy by way of self-esteem. In other words, adults who have been more severely mistreated as children tend to have lower self-esteem, which then contributes to increased envy. Previous studies have demonstrated that individuals with lower self-esteem tend to be more highly motivated to make social comparisons (Wood and Lockwood, 1999); negative upward social comparisons represent the prior condition for eliciting envy (Parrott and Smith, 1993; Smith et al., 1999; Cikara and Fiske, 2013). It therefore seemed reasonable to assume that lower self-esteem would significantly predict higher levels of envy in this study. Furthermore, previous studies have demonstrated that higher levels of child maltreatment can significantly and positively predict lower self-esteem (Stein et al., 2002; Karakuş, 2012). Accordingly, it is unsurprising that esteem plays a mediating role in the relationship between child maltreatment and envy.

However, the final model in this study did not support the mediating role of perceived social support in the relationship between child maltreatment and dispositional envy. Instead, it suggested that perceived social support plays a critical role via another significant path: child maltreatment → perceived social support → self-esteem → dispositional envy. Individuals who have suffered from early childhood maltreatment tend to have lower levels of perceived social support, which then leads to lower self-esteem, which in turn leads to increased envy. In other words, perceived social support is not a direct mediator in the relationship between child maltreatment and envy, although it plays an important role by mediating this relationship. Although lower perceived social support may not be related to higher envy directly, it may affect self-conscious emotional envy by influencing self-perceptions, such as self-esteem.

The mediating effect of perceived social support is in line with previous findings. Child physical abuse has been negatively associated with perceptions of support from family and friends and is generally related to lower levels of perceived social support in adulthood (Runtz and Schallow, 1997; Crouch et al., 2001; Vranceanu et al., 2007; Sperry and Widom, 2013). Students with lower perceived levels of social support from their classmates are more likely to have lower levels of self-esteem and higher levels of depression (Wolchik et al., 1989; Tam et al., 2011). Numerous empirical studies have shown that higher perceived social support is positively correlated with high self-esteem (Hoffman et al., 1988; Tam et al., 2011; Kong et al., 2012b; Kong and You, 2013). The mediating effect of self-esteem is in line with previous findings. In addition to the above studies on perceived social support and self-esteem, low self-esteem is theoretically (Tesser, 1988; Rosenberg et al., 1989; Salovey and Rodin, 1991; Wayment and Taylor, 1995; Staub, 1999; Marsh et al., 2001) and empirically (e.g., Rentzsch et al., 2015) related to experiencing more envy.

In the test of gender differences, we found no difference in levels of early childhood maltreatment, self-esteem, or dispositional envy. However, women had higher levels of perceived social support than men, consistent with previous studies (Antonucci and Akiyama, 1987; Kendler et al., 2005). In addition, the final model did not differ by gender, indicating that men and women share the same mechanism underlying the relationship between child maltreatment and dispositional envy. We found that the negative effect of early childhood maltreatment on perceived social support among men was significantly greater than among women. This may, in part, reflect the fact that women are more resistant to physical pain than men. Compared to men, women experience more life events and are more sensitive to them, a factor that generally increases pain perception (Aneshensel, 1992). Ramírez-Maestre et al. (2004) have found that women are better adapted to chronic pain, given similar levels of depression and anxiety in men and women. In addition, women show higher levels of resilience (Gaugler et al., 2007), recovering more easily than men from the negative effects of child maltreatment. This may reflect the effect of gender role differences on the negative effect of perceived social support in the context of childhood maltreatment.

The present study has several important limitations. First, the temporal sequence of the independent variables, mediator, and dependent variables cannot be verified, due to the correlational, cross-sectional nature of the study. Longitudinal or experimental studies would provide a more accurate assessment of causality. Second, the data were collected via self-report measures, which are subjective and vulnerable to bias, such as social desirability bias. Although scales with good reliability and validity were selected, there was no way to completely avoid self-reporting biases. Using multiple methods of evaluation (e.g., parents, peer reports) could reduce the influence of subjectivity in future studies. Third, this sample was drawn from two universities in China and the gender balance was suboptimal, which may limit the generalizability of our findings, particularly to other age groups, ethnicities, and geographic locations. The last point is that, although the Cronbach’s alpha for one subscale was relatively low, it can be accepted to some extent. Further research would provide more evidence of psychometric properties in Chinese samples.

Despite these limitations, the present study makes several important contributions. We found that early childhood maltreatment had a direct positive correlation with dispositional envy in adolescents, a subject not covered by previous studies. Furthermore, this study provides a better understanding of the mediating role of self-esteem and perceived social support in this relationship. The present findings may reflect cultural differences; future researchers are advised to use cross-cultural samples to confirm the findings. Considering the probable mechanisms, these findings may provide valuable guidance on ways of implementing positive psychological interventions aimed at reducing the negative effect of early childhood maltreatment on dispositional envy in adulthood. Helping those affected to gain social support from others and to enhance their self-esteem may work as a preventive therapy to help individuals reduce tendencies toward dispositional envy. However, research is needed to further explore the relationship between child maltreatment and different types of envy (malicious envy and benign envy) in order to propose more targeted interventions, as malicious envy can harm both physical and mental health, while benign envy can help people improve themselves. Moreover, although no significant differences were observed in the final model, gender differences within some structural paths suggest that distinct types of positive psychology interventions for child maltreatment among men and women could be considered for use in psychological services.

## Author Contributions

YX and WW have contributed equally to this work. They contributed to the experimental design, data analysis, and writing of the introduction and discussion. FG contributed to writing the data analysis and proposing amendments and addenda to the first draft. All authors approved the final version of the manuscript for submission.

## Conflict of Interest Statement

The authors declare that the research was conducted in the absence of any commercial or financial relationships that could be construed as a potential conflict of interest.
